# Evaluation of Inoculum Preparation for Etest and EUCAST Broth Dilution to Detect Anidulafungin Polyresistance in Candida glabrata

**DOI:** 10.1128/aac.00168-22

**Published:** 2022-07-11

**Authors:** Miriam Alisa Knoll, Eldina Samardzic, Wilfried Posch, Cornelia Lass-Flörl

**Affiliations:** a Institute of Hygiene and Medical Microbiology, Medical University of Innsbruck, Innsbruck, Austria

**Keywords:** *Candida*, antifungal susceptibility testing, antifungal resistance, polyresistance, inoculum preparation

## Abstract

The influence of inoculum preparation in EUCAST broth dilution and Etest to detect the coexistence of resistant and susceptible *Candida* subpopulations (defined as polyresistance [PR]) was evaluated. Cocultures of two echinocandin-resistant and susceptible clinical C. glabrata strains were used to simulate the occurrence of mixed populations in clinical samples, and antifungal susceptibility testing was performed with standard and modified approaches of inoculum preparation. Polyresistant results manifested as microcolonies or double ellipses in Etest and in single reduced optical density (OD) values (dip in OD) in microdilution. The strict inclusion of five distinct colonies of 1:5 and 1:10 resistant and susceptible cocultures led to higher rates of PR and R results compared to including one to two colonies in inoculum preparation (30% and 26% for Etest and broth dilution, respectively). Modifying the inoculum preparation by increasing the turbidity from a 2 to a 4 McFarland standard before redilution to a 0.5 McFarland standard reliably enabled the detection of resistance, with better identification of PR by Etest than by broth dilution (82% versus 32%, respectively) and of resistant minimum inhibitory concentration (MIC) values in 18% of Etests and 67% of microdilutions. The highest identification of PR succeeded with Etest and a modified 3 McFarland standard approach of inoculum preparation. Our data demonstrate that inoculum preparation as recommended and practiced does not reliably identify resistant subpopulations in polyresistant *Candida* cultures. By increasing the inoculum size for Etest assays from a 2 to a 4 McFarland standard with subsequent redilution, we propose a simple adaptation to increase reliability.

## INTRODUCTION

Early and adequate antifungal treatment is crucial for patient survival in invasive candidiasis (1), and despite advances in modern medicine and diagnostics, therapeutic failure in candidemia is still common. This is reflected by high mortality rates in candidemia, which have been assessed to reach up to 40% (2).

Polymicrobial and polyclonal infections involving *Candida* species have been described as complications for the patient management of invasive *Candida* infections (3–5). The *Candida* flora of different nonsterile body regions was suggested to be dynamic and was repeatedly found to consist of different strains and diversifying populations with numerous single nucleotide polymorphisms evolving from a common progenitor (6). Based on next-generation multi-locus sequence typing, it was shown that 12% of clinical patient samples contained two unrelated *Candida* strains (7), suggesting a considerable within-host diversity of *Candida* infections.

Guidelines for antifungal susceptibility testing (AFST) (CLSI, EUCAST) and user manuals for commercially available minimum inhibitory concentration (MIC) test strips recommend preparation of the inoculum by, including five or several colonies above 1 mm in diameter (8–10), respectively. All methods use a cell density of 0.5 McFarland standard, which can be obtained by collecting a small number of colonies. If susceptible and resistant genotypes are involved in an infection, common methods for routine AFST may not be able to reflect this clinical situation, as their results are based on arbitrarily selected single colonies (11). Furthermore, it was shown that with a standard inoculum preparation, resistant subpopulations are spotted more efficiently with Etest assays than with EUCAST broth dilution (11).

**FIG 1:**
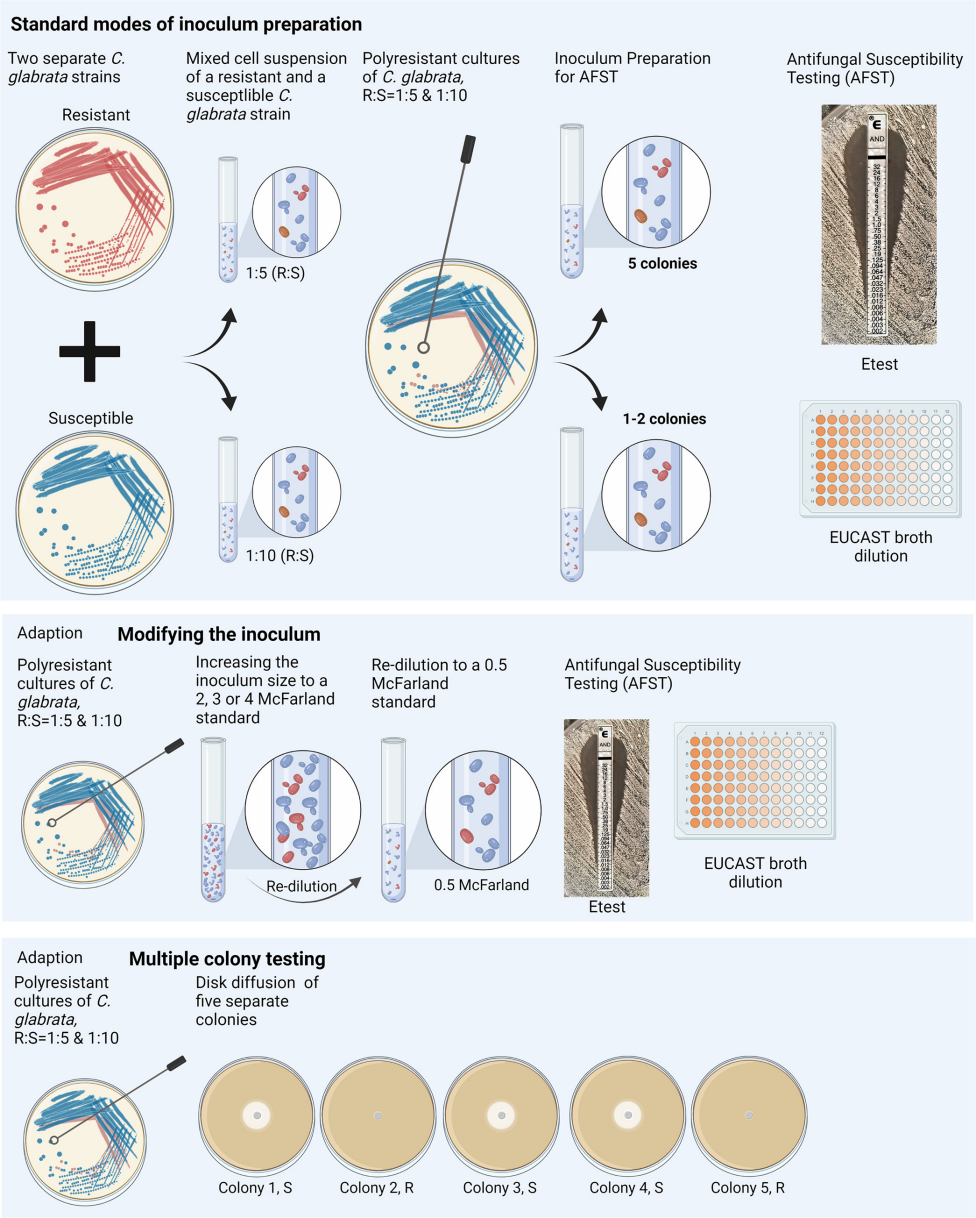
Visualization of AFST by EUCAST broth dilution and Etest performed on a coculture of a susceptible strain (S, blue) and a smaller proportion of a resistant strain (R, red) of the same *Candida* species. R and S strains of the same *Candida* species were mixed in defined proportions (1:5 and 1:10, R:S, respectively) to simulate a polyresistant culture from a patient sample. Five colonies were used for the inoculum for AFST and were rediluted to a 0.5 McFarland standard. The second approach was performed by adding one to two colonies until the recommended turbidity of a 0.5 McFarland standard was reached. Adaptions to increase the detection of polyresistance (PR): modifying the inoculum by suspending a higher number of cells via a higher initial McFarland standard with subsequent redilution for testing increases the probability of including all present phenotypes. Disk diffusion screening by disk diffusion testing of five separate colonies from the polyresistant culture. One diverging result indicates PR. Made in BioRender (biorender.com).

In this study, we investigated if the mode of inoculum preparation for AFST with Etest and EUCAST broth dilution (microdilution) has an impact on the detection of echinocandin polyresistance (PR, same species but with a mixed resistant and susceptible population) in artificial C. glabrata cocultures containing 10 and 20% anidulafungin-resistant subpopulations (Fig. 1 and Table 1).

**TABLE 1:** Characteristics of strains used to simulate polyresistant *Candida* cultures

Strain ID	Species	MIC anidulafungin (μg/mL)		*Fks* mutation
		MIC EUCAST	MIC Etest	
1S	C. glabrata	0.03	0.016	No mutation
2S	C. glabrata	0.016	0.016	No mutation
1R	C. glabrata	4	8	S663P
2R	C. glabrata	4	4	S629P

## RESULTS

**FIG 2:**
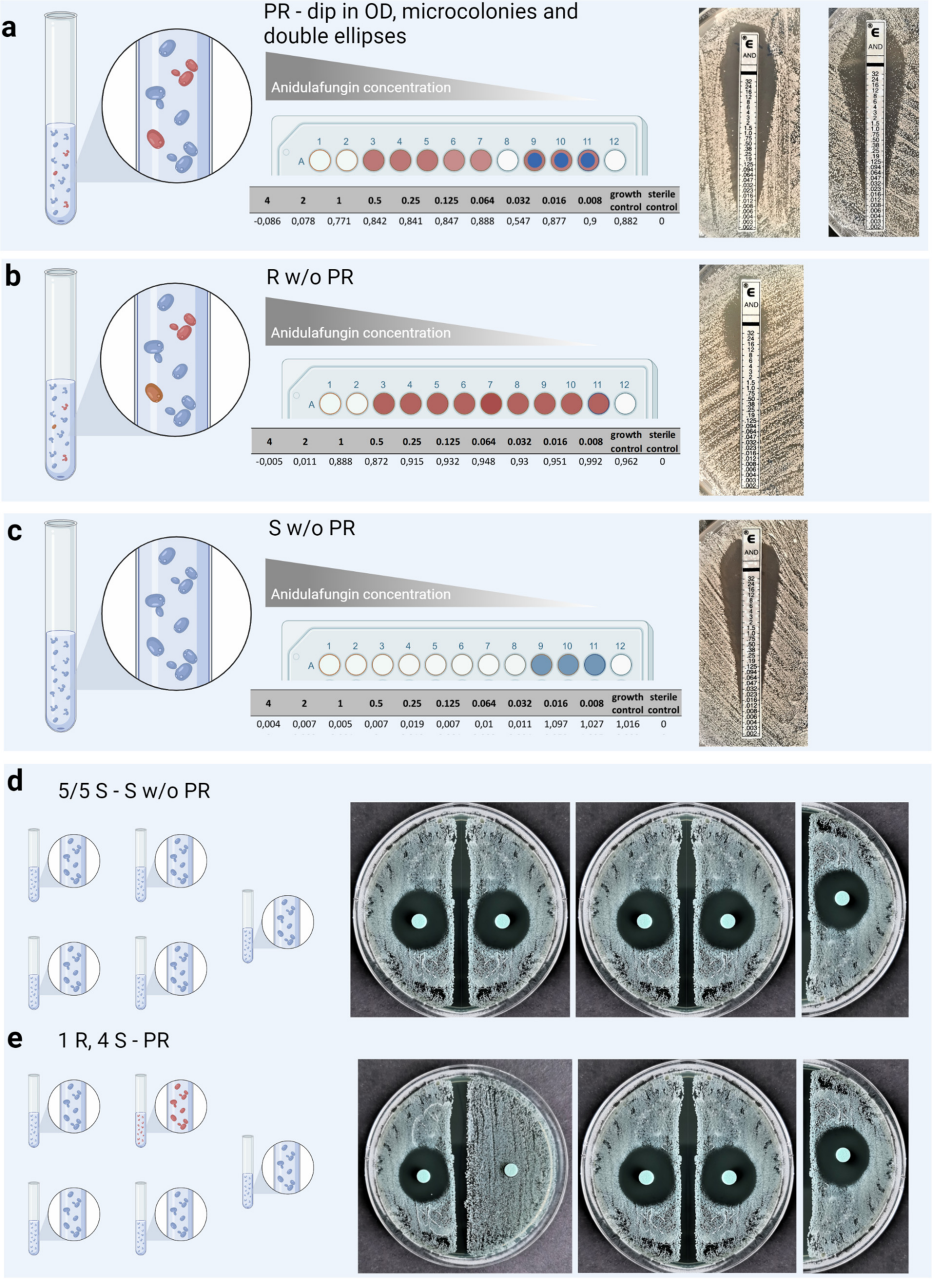
Results of AFST by broth dilution, Etest, and disk diffusion screening on a PR C. glabrata culture. (a) PR by random inclusion of cells from both strains. Results of broth dilution and photometrically measured optical density (OD) suggest PR by a dip in OD. A dip is defined as an isolated reduced value below 75% OD of the growth control. Etest results suggest PR by microcolonies growing within the ellipse of inhibition or a double ellipse. (b) Resistant results without signs of PR (R w/o PR) by random inclusion of cells from both strains. Results of broth dilution with photometrically measured OD and Etest results show homogeneously resistant results. (c) Susceptible results without signs of PR (S w/o PR) by random suspension of colonies deriving only from the susceptible strain. Results of broth dilution with photometrically measured OD and Etest results show homogeneously susceptible results. (d) Five out of five S results without signs of PR (S w/o PR) by disk diffusion screening of a random selection of colonies deriving only from the susceptible strain. (e) PR by one diverging R result out of five tests by disk diffusion screening of a random inclusion of cells from both strains. Made in BioRender (biorender.com).

**FIG 3:**
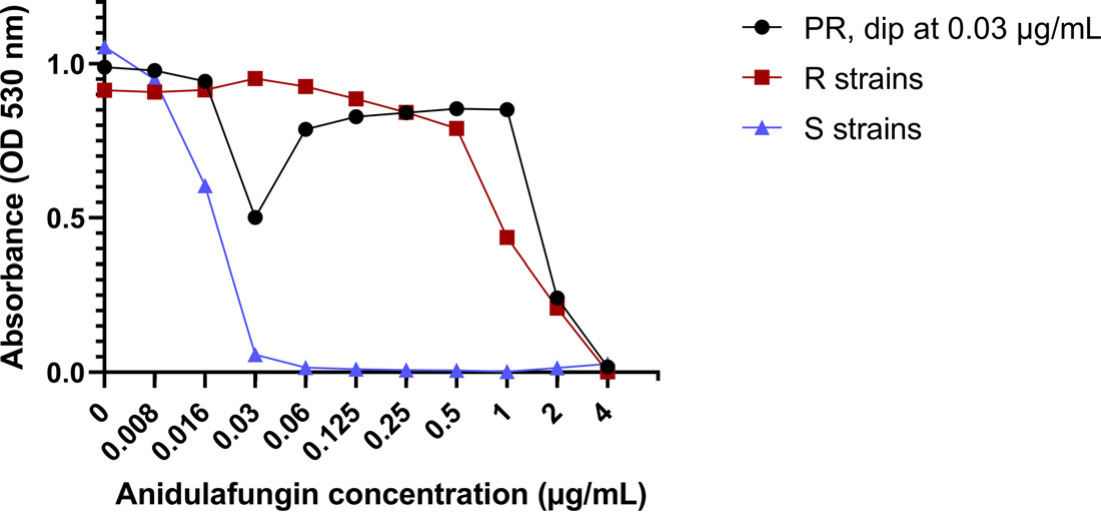
Illustration of the observed dip in the optical density (OD) of polyresistant (PR) cultures. Concentration-absorbance curves for the mean ODs of PR cultures exhibiting a dip in OD compared to the pure resistant and susceptible strains. A dip in OD was arbitrarily defined as a reduction of the OD value around the MIC of the susceptible strain by at least 25%, relative to the growth control, without subsequent growth inhibition below 50%. OD was measured using EUCAST spectrophotometric reading after 24 h of incubation.

The reading of AFST results included an inspection for signs of PR, such as a dip in optical density (OD) for microdilution (Fig. 2 and 3) or double ellipses and microcolonies in Etest (Fig. 2). AFST results were read as susceptible without signs of PR (S w/o PR), resistant without signs of PR (R w/o PR), or PR.

### Standard methodology of inoculum preparation.

From 1:5 and 1:10 (R:S) polyresistant cocultures, Etest and EUCAST microdilution were performed with 1 to 2 colonies and with 5 colonies. These are commonly used modes of inoculum preparation, with the former coming closer to the real-life situation in clinical laboratories and the latter being recommended by the guidelines (9, 10). Identification of polyresistance (PR) was successful in 15% of the microdilution assays with 5 colonies and in 5% of the microdilution assays with 1 to 2 colonies (Table 2). Microdilution assays with resistant MIC values were classified as polyresistant if they showed a dip in OD at 0.03 or 0.016 mg/L (Fig. 2). The R subpopulation was revealed with R w/o PR in 21% (1 to 2 colonies) and 35% (5 colonies), while S w/o PR were obtained in 74% (1 to 2 colonies) and 50% (5 colonies) of microdilutions. Higher detection rates were obtained by Etest, in which double ellipses or microcolonies indicated PR (Fig. 2). In Etest, the inclusion of 5 colonies for inoculum preparation led to a PR identification rate of 30% compared to 5% with 1 to 2 colonies. With 5 colonies, the overall rate of S w/o PR was equal between Etest and microdilution (50%) while the rate was higher for 1 to 2 colonies (70% for Etest, 74% for microdilution). Slightly higher rates of S w/o PR were observed for tests with a 1:10 (R:S) dilution than for those with a 1:5 (R:S) dilution (Table S1). No difference in results was observed between translating the Etest MICs to EUCAST breakpoints, applying Etest ECVs (12, 13) and previously used MIC cutoff values for Etest to classify isolates as resistant (14). Altogether, the capability of common methods to detect PR was limited, especially when broth dilution was used.

**TABLE 2:** Results of AFST by broth dilution, Etest, and disk diffusion screening of a PR C. glabrata culture in percent^*a*^

Intervention	Turbidity	Method	AFST performed on PR cultures, results of AFST in %		
			PR (%)	R w/o PR (%)	S w/o PR (%)
Standard methodology	5 colonies	Etest (*n* = 20)	30	20	50
		Microdilution (*n* = 20)	15	35	50
	1 to 2 colonies	Etest (*n* = 20)	5	25	70
		Microdilution (*n* = 20)	5	21	74
Modifying the inoculum	McFarland 2	Etest (*n* = 20)	75	25	0
		Microdilution (*n* = 20)	40	60	0
	McFarland 3	Etest (*n* = 20)	95	5	0
		Microdilution (*n* = 20)	30	65	5
	McFarland 4	Etest (*n* = 20)	75	25	0
		Microdilution (*n* = 20)	25	75	0
	Total	Etest (*n* = 60)	82	18	0
		Microdilution (*n* = 60)	32	67	2
Screening		Disk diffusion (*n* = 40)	65	0	35

a AFST results were read as susceptible without signs of PR (S w/o PR), resistant without signs of PR (R w/o PR), or polyresistant (PR). In disk diffusion screening, the detection of at least one diverging screening result out of five tests was classified as PR, while the detection of five out of five S screening results was defined as S w/o PR. Five colonies were used for the inoculum for AFST and rediluted to a 0.5 McFarland standard. The second approach is performed by adding one to two colonies until the recommended turbidity of a 0.5 McFarland standard is reached. Adaptions to increase the detection of PR, modifying the inoculum: the suspension of a higher number of cells by producing a higher initial McFarland standard with subsequent redilution for testing increases the probability of including all present phenotypes. Disk diffusion screening was performed via the testing of five separate colonies from the PR culture. One diverging result indicates PR.

### Modifying the inoculum.

To increase the probability of including colonies of the R subpopulation, tests were performed with a higher initial inoculum size of 2, 3, and 4 McFarland standard prior to redilution to 0.5 for testing (Fig. 1). PR was identified with the Etest method in 75% to 95%, with the highest detection rates at an initial inoculum size of 3 McFarland standard (95%) (Table 2). PR identification with Etest resulted in a double ellipse in 73% and microcolonies in 27% (Fig. 2). R w/o PR was obtained in 5% to 25% of Etests, and none of the Etests with increased inoculum sizes resulted in S w/o PR. Contrarily, increasing the number of colonies did not lead to the same level of PR identification in broth dilution. However, this increase minimized S w/o PR to 2% overall. In 60% to 75% of the broth dilutions, using increased McFarland standards, R w/o PR was found, while PR identification was achieved in 25% to 40%. PR identification manifested as a dip in OD at the MIC value of the S strain (Fig. 2). Results shifted toward R w/o PR with increasing inoculum sizes, with the highest identification of PR at a 2 McFarland standard initial inoculum (40%) and the highest rate of R w/o PR at a 4 McFarland standard initial inoculum (75%). Overall, modified inoculum preparation led to a cumulative PR identification rate of 82% for Etests and 32% for microdilutions. R w/o PR was achieved in 18% of Etests and 67% of microdilutions, and S w/o PR was observed in 0% and 2% for Etest and microdilution, respectively. No significant difference was observed between the dilution ratios (1:10 or 1:5, R:S) (Table S1), and no difference in results was observed between translating the Etest MICs to EUCAST breakpoints, applying Etest ECVs (12, 13) and tentative Etest breakpoints (14) for categorization. Altogether, increasing the turbidity of the inoculum led to almost complete identification of PR and detection of the R subpopulation.

### Disk diffusion screening.

Last, disk diffusion screening was performed to evaluate the PR identification rates of separate multiple colony testing (Fig. 1). The separate screening of 5 single distinct colonies led to a 65% identification of PR, whereas 35% of colonies of the susceptible strain were identified (S w/o PR; Table 2).

Results are summarized in Table 2 and Fig. 2. Overall, Etest enabled higher rates of PR detection than did microdilution, while the rate for S w/o PR was similar between both methods. However, PR identification rates varied greatly between the different modes of inoculum preparation. Including 5 colonies resulted in less S w/o PR than using only 1 to 2 colonies. Identification of PR by microdilution resulted in a dip in OD, while in Etest, PR identification manifested as double ellipses or microcolonies. Increasing the inoculum size to a 2 to a 4 McFarland standard minimized S w/o PR for both Etest and microdilution and resulted in higher PR identification rates for Etest than for microdilution.

## DISCUSSION

Performing AFST on polyresistant cultures with different proportions of R subpopulations showed that the capability of common methods for AFST to detect heterogeneous MICs is limited. Infections due to PR have hitherto not been regarded as a clinically significant problem; however, the need for a reliable detection method is apparent. Polyclonal infections have been observed for catheter-related cases of candidemia due to both infection with different species of *Candida* as well as infection with unrelated clones of the same *Candida* species (4, 5). Recent data showed a certain within-host genomic diversity of *Candida*, describing a coexistence of several unrelated strains and progenitors diverging into distinct lineages within a host (6, 7, 15). Of note, these findings are distinct from the phenomenon of heteroresistance, where, usually, an even smaller proportion of a monoclonal strain exhibits unstable resistance phenotypes (16, 17). Data are still scarce on the diverse types of heteroresistance and discordant populations in fungi. Early findings indicate high rates of heteroresistance for C. glabrata and fluconazole, while no significant levels were found for echinocandins (18). Heteroresistance does not refer to the presence of two distinct strains but describes cell-to-cell variability on a single-strain level (18). In more detail, heteroresistance refers to isogenic populations with small subpopulations of resistant cells (17), while polyresistance refers to the phenotypical variability in clinical AFST, including the presence of two distinct strains. In a recent study, we found that upon separate testing of single colonies, heterogeneous MICs from same-species clinical *Candida* cultures occurred in 8% of clinical samples (19). MLST and RAPD did not reveal a uniform genetic correlate in the strains studied but rather suggested a phenotypical expression of genomic variability. This is in line with previous studies, where next-generation multi-locus sequence typing showed that 12% of patient samples contained two unrelated *Candida* strains (7). Polyresistance may also include heteroresistance but is not limited to any such underlying mechanism. To avoid interference with the already existing and defined term, heteroresistance, we created a new expression to help describe the phenotypical heterogeneity of results often encountered in clinical practice. However, some similarities to heteroresistance in the pitfalls of AFST can be assumed, as using the MIC value as the sole measure of susceptibility does not represent heterogeneous microbial infections.

For our investigation regarding the presence of two distinct C. glabrata strains, we estimated proportions of the R strains with concentrations of 1:5 and 1:10, relative to the susceptible population. These ratios were chosen based on results of next-generation multi-locus sequence typing, which revealed the presence of two distinct *Candida* strains in patient samples, with proportions of the less frequent strain ranging from 18% to 47% (average 31% ± 13%) (7). We selected proportions of 1:5 (20% frequency) and 1:10 (10% frequency) to cover the lower range of these real-life ratios. However, smaller proportions could be possible in infection sites, and this case has not yet been covered in the literature. PR identification rates were low for both Etest and microdilution when regular inoculum preparation was performed. Failure of PR detection might lead to discordant results upon repeated testing of clinical *Candida* samples and may contribute to the burden of unexplained therapeutic failure.

Etest achieved better identification of PR than microdilution. The occurrence of double ellipses or microcolonies (Fig. 2b) more apparently indicates PR than does the subtler dip in OD observed in microdilution, which was defined as an isolated reduced value below 75% OD, relative to the growth control. Similar observations have been described in Etests directly performed on positive blood cultures, where the presence of *fks* mutants together with echinocandin-susceptible strains led to the appearance of double ellipses (11). Our findings suggest that inversely, these MIC phenomena occurring in clinical samples may be caused by PR. As targeted attention is required for the identification of a dip in OD, these MIC phenomena may be overlooked in clinical practice. Additionally, the difficulty in the correct interpretation of microdilution MIC phenomena represents a limitation compared to Etest, where a double ellipse or colonies within the zone of inhibition quite instinctively indicate a PR profile. However, the Etest Reading Guide recommends ignoring such microcolonies (20); hence, further studies are necessary to clarify this issue.

Microdilution showed S w/o PR in 74% when performed with the 1 to 2 colonies approach, and even with 5 colonies, 50% of the tests resulted in S w/o PR. The Etest method produced higher PR identification rates due to the appearance of microcolonies that were otherwise uncommon for echinocandins and double ellipses that quite clearly indicated the presence of two distinct resistance profiles. While in practice, the inoculum is often prepared by adding one or two colonies until the desired turbidity is reached, guidelines and instructions advise to include at least five colonies before redilution to the desired turbidity equivalent. This results in a slightly higher workload compared to a McFarland oriented approach, as the inclusion of five distinct colonies most likely requires a redilution of the suspension. However, adhering to a five colonies approach also increases the probability of including a putative subpopulation. This principle was effective in both broth dilution and Etest.

A further decrease of S w/o PR was achieved by increasing the initial inoculum size. Regarding the optimal initial inoculum size, the rate of R w/o PR increased with higher turbidity (up to 75% for microdilutions with a 4 McFarland standard). The high number of viable cells from the R subpopulation most likely produces sufficient growth above the MIC of the susceptible strain to mimic a homogeneous R strain. In conclusion, broth dilution is not the optimal method to detect PR, even with adaptions or targeted attention. However, if the goal is the detection of R subpopulations, the proposed modification of inoculum preparation elevates detection rates, independent of the testing method.

Increasing the McFarland standard for Etests led to almost complete identification of PR. Hereby, the highest PR identification rates were reached at a 3 McFarland standard turbidity, with 95% PR identification, while in the remaining 5%, R w/o PR was obtained. These observations are in line with investigations regarding bacteria, where a modified Etest achieved detection of heteroresistance if applied directly from the sample; however, this detection was not achieved if the strain was subcultured before testing (21). Similarly, directly performing Etests on positive blood cultures spiked with echinocandin-resistant and susceptible C. glabrata strains showed better identification of PR than did performing EUCAST microdilution on 24 h cultures (11). Our results confirm that performing AFST with standard inoculum preparation does not reliably identify PR. While in clinical practice, direct AFST is often not possible, we offer a simple adaption to still ensure reliable detection of R subpopulations by increasing the initial inoculum size.

Last, separate multiple colony testing with a disk diffusion test was evaluated as a priceworthy alternative to screen for PR. The assumption was that including 5 colonies in one inoculum would deliver similar results as the separate testing of five distinct colonies, since, ultimately, the same number of colonies was included. However, this was not the case, and more than twice the PR identification rate compared to the single test result was obtained. Nevertheless, the results are not completely comparable, as in the direct competition of S and R strains within one plate or microdilution well, the relative growth of the R strain may be reduced. Compared to one separate test per colony without competition between the strains, PR expression might be not as pronounced in single tests with a higher inoculum. Also, regarding S w/o PR rates, better results were obtained by separate multiple colony screening (35% S w/o PR versus 50% for 5 colonies in broth dilution and Etest). However, in light of the 35% S w/o PR, the additional workload of multiple colony testing does not seem justified.

Altogether, Etest enabled a better display of PR infections than did broth dilution. Identification of PR by microdilution resulted in a dip in OD, which suggested that, inversely, MIC phenomena occurring in clinical samples may be caused by PR. In Etests, PR identification manifested as double ellipses or microcolonies. The number of colonies used for inoculum preparation directly influenced PR identification rates. By further increasing the inoculum size from a 2 to a 4 McFarland standard and only then rediluting the solution to the required turbidity equivalent for testing, we offer a simple and feasible adaption for AFST inoculum preparation to ensure the reliable detection of R subpopulations in a 1:5 to 1:10 proportion. Further studies are needed to address the applicability of our proposed adaptions to inoculum preparation to a wider range of *Candida* species and antifungals.

## MATERIALS AND METHODS

Two clinical *fks* wild-type isolates susceptible to anidulafungin and two *fks* hot spot mutant C. glabrata isolates resistant to anidulafungin were included in the study (Table 1). All isolates were previously analyzed and sequenced (22). Isolates were subcultured on Sabouraud dextrose agar twice before testing. Species were confirmed by matrix-assisted laser desorption ionization-time of flight mass spectrometry (MALDI-TOF MS) Biotyper (Bruker Daltonik GmbH, Bremen, Germany). MICs of anidulafungin against the strains were determined by Etest (bioMérieux, Marcy l’Etoile, France) and the EUCAST broth dilution method (E.Def 7.3.2) (Table 1). Anidulafungin was selected as the marker of echinocandin resistance, as it was shown to identify *fks* hot spot mutants more reliably than the other echinocandins (22). MICs for anidulafungin were in the susceptible category for the two wild-type strains and in the resistant category for the *fks* hot spot mutant strains.

Using 1:5 and 1:10 ratios of R and S strains as the inoculum for Etest, we observed double ellipses and microcolonies as growth patterns of the S and R strains. Using 1:5 and 1:10 ratios (R:S, respectively) as the inoculum for EUCAST microdilution, we observed MICs in the R category with a dip in OD. A dip in OD was defined as a reduction of the OD value around the MIC of the susceptible strain by at least 25%, relative to the growth control, without subsequent growth inhibition below 50% (Fig. 3). The term, dip in OD, was arbitrarily defined. These AFST phenomena (double ellipses, microcolonies in Etest, dip in OD in microdilution) were defined as detection of PR.

As one of the underlying mechanisms for PR, cocultures of susceptible and resistant strains were simulated by mixing two combinations of anidulafungin R and S strains in ratios of 1:5 and 1:10 (R:S, respectively, Fig. 1) prior to streaking Sabouraud plates from these suspensions. From each mixed cell suspension, 35 full Sabouraud dextrose agar plates were streaked and incubated for 24 h at 37°C. These cultures, containing morphologically identical R and S C. glabrata strains, represented seemingly homogeneous cultures in clinical practice. The influence of inoculum preparation on the identification rates of PR was evaluated.

### Standard methodology of inoculum preparation.

**(i) Five colonies.** Inocula for AFST with anidulafungin were prepared as recommended in the manufacturers’ instructions for Etest as well as the EUCAST guidelines. This workflow included five distinct colonies and a subsequent redilution to a 0.5 McFarland standard. Five separate attempts per strain combination and dilution ratio were conducted and used as the inoculum for Etest and microdilution with anidulafungin (*n* = 20).

**(ii) One to two colonies.** Colonies were added only until the recommended turbidity of 0.5 McFarland was reached, which usually occurred after the suspension of one or two colonies from the 24-h Sabouraud culture. Hence, this workflow included fewer colonies than the number recommended by guidelines but may better reflect the real-life situation encountered in diagnostic laboratories. Five separate AFST attempts per strain combination and dilution ratio were conducted and used as the inoculum for Etest and microdilution with anidulafungin (*n* = 20).

### Modifying the inoculum.

To increase the probability of including colonies of the resistant strain, tests were performed with increased initial inoculum sizes of 2, 3, and 4 McFarland standard turbidity. Distinct colonies as well as streaks through the culture were used to obtain the higher inoculum sizes. The suspensions were then rediluted to a 0.5 McFarland standard for the inoculation of Etest and EUCAST microdilution. Five separate attempts per strain combination and dilution ratio were conducted for each modified McFarland standard and were used as the inoculum for Etest and microdilution with anidulafungin (*n* = 60).

### Multiple colony testing.

Multiple colony testing refers to the separate AFST of usually five distinct colonies from a *Candida* culture (19, 23) and was performed here by an anidulafungin disk diffusion assay (24, 25). Empty 6 mm sterile paper disks were impregnated with 20 μL containing 2 μg anidulafungin, 1% dimethyl sulfoxide (DMSO), and 0.1% polysorbate 80 (Tween 80) and were then left to dry at room temperature. From the 24 h cocultures, five well-isolated colonies ≥1 mm in diameter were brought in separate 0.5 McFarland standard solutions and streaked in three directions on Mueller-Hinton agar plates supplemented with 2% glucose and 0.5 μg/mL methylene blue. The prepared anidulafungin disks were placed after letting the inoculated plates dry for 15 min. Ten separate attempts per coculture and dilution ratio were conducted (*n* = 40).

All methods and modifications are visualized in Fig. 1.

### Interpretation of results.

Strains were classified as susceptible or resistant according to EUCAST breakpoints (26). Broth dilution was read photometrically by a microplate reader (Tecan, Männedorf, Switzerland) at 530 nm. Etest MICs were translated to EUCAST breakpoints (26) as well as Etest ECVs and tentative Etest breakpoints (14) for categorization.

Reading of results included the inspection for signs of PR, such as a dip in OD for microdilution or double ellipses and microcolonies for Etest. AFST results were read as susceptible without signs of PR (S w/o PR), resistant without signs of PR (R w/o PR), or PR.

In the disk diffusion assay, the numbers of R and S results per coculture were documented. The detection of at least one diverging screening result out of five tests was classified as PR. Five out of five R disk diffusion screening results were defined as R w/o PR. Five out of five S disk diffusion screening results were defined as S w/o PR.

Visualizations of the results are shown in Fig. 2. Fig. 1 and 2 have been created using BioRender.com. Fig. 3 has been created using GraphPad Prism version 9.3.1 for Windows, GraphPad Software, San Diego, California, USA, www.graphpad.com.
