# Supplementary material for: Evaluation of Inoculum Preparation for Etest and EUCAST Broth Dilution to Detect Anidulafungin Polyresistance in Candida glabrata
Source: Antimicrob Agents Chemother. 2022 Jul 11;66(8):e00168-22. doi: 10.1128/aac.00168-22 (PMC9380570; doi:10.1128/aac.00168-22)
Supplement: Supplemental file 1 — Table S1. Download aac.00168-22-s0001.pdf, PDF file, 0.5 MB [file aac.00168-22-s0001.pdf]

Supplementary Material

Table 1 Results of AFST by broth dilution, Etest© and disk diffusion screening of a polyresistant (PR) *C. glabrata* culture by dilution ratio (1:10 or 1:5, resistant (R) to susceptible (S)). AFST results were read as susceptible without signs of PR (S w/o PR), resistant without signs of PR (R w/o PR) or polyresistant (PR). In disk diffusion screening, the detection of at least one diverging screening result of five tests was classified as PR, while S w/o PR was defined as five out of five S screening results. Standard modes of inoculum preparation were performed by including five distinct colonies in the inoculum for AFST and re-diluting to a 0.5 McFarland standard for AFST, and by adding one to two colonies until the recommended turbidity of 0.5 McFarland for AFST was reached. **Adaptions** to increase the detection of polyresistance: **Modifying the inoculum**: The suspension of a higher number of cells by producing a higher initial McFarland standard with subsequent re-dilution for testing increases the probability of including all present phenotypes. **Multiple colony testing** by disk diffusion for five separate colonies from the polyresistant culture. Already one diverging result indicates polyresistance. Results are displayed as the number of tests obtaining the respective result (n) and percentage per column. One McF-IP broth dilution test was excluded due to insufficient growth.

**Agar based methods**		Standard methodology		Modifying the inoculum			Screening
**Results**		**1-2 colonies**	**5 colonies**	**McFarland 2**	**McFarland 3**	**McFarland 4**	**Multiple colony testing**
**PR**	1 to 10 (n)	1	4	9	9	8	13
	1 to 5 (n)	0	2	6	10	7	13
	Altogether (n)	1	6	15	19	15	26
	Percentage	**5%**	**30%**	**75%**	**95%**	**75%**	**65%**
**R w/o PR**	1 to 10 (n)	1	0	1	1	2	0
	1 to 5 (n)	4	4	4	0	3	0
	Altogether (n)	5	4	5	1	5	0
	Percentage	**25%**	**20%**	**25%**	**5%**	**25%**	**0%**
**S w/o PR**	1 to 10 (n)	8	6	0	0	0	7
	1 to 5 (n)	6	4	0	0	0	7
	Altogether (n)	14	10	0	0	0	14
	Percentage	**70%**	**50%**	**0%**	**0%**	**0%**	**35%**
	n total	20	20	20	20	20	40

**Microdilution**		Standard methodology		Modifying the inoculum		
**Results**		**1-2 colonies**	**5 colonies**	**McFarland 2**	**McFarland 3**	**McFarland 4**
**PR**	1 to 10 (n)	0	0	5	3	2
	1 to 5 (n)	1	3	3	1	0
	Altogether (n)	1	3	8	4	2
	Percentage	**5%**	**15%**	**40%**	**20%**	**10%**
**R w/o PR**	1 to 10 (n)	1	4	5	7	8
	1 to 5 (n)	3	3	7	8	10
	Altogether (n)	4	7	12	15	18
	Percentage	**21%**	**35%**	**60%**	**75%**	**90%**
**S w/o PR**	1 to 10 (n)	9	6	0	0	0
	1 to 5 (n)	5	4	0	1	0
	Altogether (n)	14	10	0	1	0
	Percentage	**74%**	**50%**	**0%**	**5%**	**0%**
	n total	19	20	20	20	20
